# Household trends in access to improved water sources and sanitation facilities in Vietnam and associated factors: findings from the Multiple Indicator Cluster Surveys, 2000–2011

**DOI:** 10.3402/gha.v9.29434

**Published:** 2016-02-29

**Authors:** Tran Thi Tuyet-Hanh, Jong-Koo Lee, Juhwan Oh, Hoang Van Minh, Chul Ou Lee, Le Thi Hoan, You-Seon Nam, Tran Khanh Long

**Affiliations:** 1 Hanoi School of Public Health, Hanoi, Vietnam; 2 JW LEE Center for Global Medicine, Seoul National University College of Medicine, Seoul, Republic of Korea; 3 Department of Family Medicine, Seoul National University College of Medicine, Seoul, Republic of Korea; 4 Environmental Health Department, Hanoi Medical University, Hanoi, Vietnam

**Keywords:** MICS, Vietnam, improved water sources, sanitation facilities

## Abstract

**Background:**

Despite progress made by the Millennium Development Goal (MDG) number 7.C, Vietnam still faces challenges with regard to the provision of access to safe drinking water and basic sanitation.

**Objective:**

This paper describes household trends in access to improved water sources and sanitation facilities separately, and analyses factors associated with access to improved water sources and sanitation facilities in combination.

**Design:**

Secondary data from the Vietnam Multiple Indicator Cluster Survey in 2000, 2006, and 2011 were analyzed. Descriptive statistics and tests of significance describe trends over time in access to water and sanitation by location, demographic and socio-economic factors. Binary logistic regressions (2000, 2006, and 2011) describe associations between access to water and sanitation, and geographic, demographic, and socio-economic factors.

**Results:**

There have been some outstanding developments in access to improved water sources and sanitation facilities from 2000 to 2011. In 2011, the proportion of households with access to improved water sources and sanitation facilities reached 90% and 77%, respectively, meeting the 2015 MDG targets for safe drinking water and basic sanitation set at 88% and 75%, respectively. However, despite these achievements, in 2011, only 74% of households overall had access to combined improved drinking water and sanitation facilities. There were also stark differences between regions. In 2011, only 47% of households had access to both improved water and sanitation facilities in the Mekong River Delta compared with 94% in the Red River Delta. In 2011, households in urban compared to rural areas were more than twice as likely (odds ratio [OR]: 2.2; 95% confidence interval [CI]: 1.9–2.5) to have access to improved water and sanitation facilities in combination, and households in the highest compared with the lowest wealth quintile were over 40 times more likely (OR: 42.3; 95% CI: 29.8–60.0).

**Conclusions:**

More efforts are required to increase household access to both improved water and sanitation facilities in the Mekong River Delta, South East and Central Highlands regions of Vietnam. There is also a need to address socio-economic factors associated with inadequate access to improved sanitation facilities.

## Introduction

Adequate access to safe drinking water and hygienic toilets are basic needs required to ensure good public health. Polluted water and lack of sanitation increases the risk of various diseases, including cholera, typhoid, schistosomiasis and some cancers through exposure to carcinogens (1). Access to clean water is important for promoting hygienic practices, such as hand and face washing, which help to reduce risks of endemic diarrhea; respiratory and skin infections; trachoma and other types of eye infections (2, 3). Globally, approximately 2.4 million deaths a year (4.2% of all deaths) could be prevented if hygiene was properly practiced, but in order to do so it is important to have adequate access to safe drinking water and basic sanitation (3).

Millennium Development Goal (MDG) number 7, target 7.C aimed to reduce the proportion of people without sustainable access to safe drinking water and basic sanitation by half between 1990 and 2015. Since 1990, over 2 billion people worldwide have gained access to improved drinking water, and almost 2 billion people have gained access to improved sanitation (4). However, currently, still more than 700 million people, mostly the poor and the marginalized in developing countries such as Vietnam, lack access to improved sources of drinking water. Of these, 173 million people still rely on untreated surface water, most of whom (over 90%) live in rural areas. There are a number of reasons for this, including high population growth in developing countries, slow rates of capital investment, and the resource challenges associated with developing effective water resources in these countries (1). Waste water management is also a huge problem for developing countries, where approximately 2.5 billion people do not use any improved toilets, and 1 billion people still practice open defecation (4).

The Vietnam Multiple Indicator Cluster Survey (MICS) is an international household survey program developed by the United Nations Children's Fund (UNICEF). The MICS was conducted in Vietnam on five occasions, in 1995, 2000, 2006, 2011, as part of the global MICS, and also in 2014 by the General Statistics Office of Vietnam, with financial and technical support from UNICEF and financial support from the United Nations Population Fund (UNFPA) (5–8). Approximately, 8,000 to nearly 12,000 households from all six regions in Vietnam were involved in each MICS. The results of the MICS provide up-to-date information on the health status of children and women and measure key indicators which allow countries to monitor progress towards the MDGs and other internationally agreed targets (5). The MICS reports show a range of indicators, including the use of improved drinking water sources and improved sanitation facilities. However, these reports do not analyze water and sanitation facilities in combination, nor trends over time or associated demographic and socio-economic factors.

This study describes household trends in access to improved water and sanitation facilities separately, and analyses factors associated with access to improved water and sanitation facilities in combination, in Vietnam from 2000 to 2011. The work is part of a collaborative project on strengthening the health system in Vietnam. The results will help to draw a comprehensive picture of water and sanitation facilities in Vietnam, and inform policy makers of some of the challenges faced in providing adequate access to improved water sources and sanitation.

## Methods

### Data source

Data were extracted from the data sets of three MICSs conducted in 2000, 2006, and 2011 in Vietnam. The MICSs were nationally representative surveys covering a broad range of issues affecting the health, development and living conditions of Vietnamese women and children. The number of households that completed the survey interviews in these MICSs was 9,117 in the year 2000; 9,473 in the year 2006; and 11,614 in the year 2011, respectively.

### Study variables

#### Dependent variables

‘Improved water sources’ consisted of piped water (into dwelling, yard/plot, and public tap/stand-pipe), tube-well/bore-hole, protected well, protected spring, and rainwater. Bottled water was only included as an improved water source if the household used another improved water source for other purposes, such as hand-washing and cooking (5). Unprotected wells, unprotected springs, tanker truck, cart with tank/drum, and surface water were considered as ‘unimproved water sources’.

‘Improved sanitation facilities’ included piped sewer systems, septic tanks, pit latrines, ventilated improved pit latrines, pit latrines with slabs, and composting toilets. ‘Unimproved sanitation facilities’ included flush/pour flush to somewhere else, flush/pour flush to an unknown place, pit latrine without slab/open pit, buckets, hanging toilets/hanging latrines, open defecation into bushes, other fields, and the absence of sanitary facilities (5).

A variable that described combinations of access to improved water sources and sanitation facilities was defined according to whether households had access to improved water sources, and/or access to improved sanitation facilities. Household trends in the use of improved water sources and sanitation facilities in combination over time were measured according to three categories: 1) both water sources and sanitation facilities were improved, which was classified as ‘improved water sources AND improved sanitation facilities’; 2) both water sources and sanitation facilities were unimproved, that is, ‘unimproved water sources AND unimproved sanitation facilities’; and 3) either water sources or sanitation facilities were unimproved, grouped in ‘unimproved water sources OR unimproved sanitation facilities’.

#### Independent variables

The independent variables included in the analysis in this article referred to geographic, demographic, and socio-economic characteristics. Geographical regions of Vietnam were divided into 1) Red River Delta, 2) Northern Midlands and Mountain areas, 3) North Central area and Central Coastal area, 4) Central Highlands, 5) South East, and 6) Mekong River Delta. Living area was divided into urban or rural. The economic condition of the household was assessed based on an asset-based wealth index, constructed using the principal component analysis (PCA) method, which is classified into wealth quintiles whereby: 1) poor, 2) near poor, 3) average, 4) fair, and 5) rich. The PCA technique is well described elsewhere (9). Ethnicity (Kinh and/or Hoa (Chinese) vs. Non-Kinh), sex, and educational level were based on the characteristics of the head of the household. Educational level was divided into three groups: 1) secondary education or higher, 2) primary education, and 3) none.

### Statistical analysis

Descriptive analyses were undertaken to describe the status and trends of access to improved water sources and sanitation facilities over time and by location, demographic, and socio-economic factors. For analytical statistics, chi-squared tests with Bonferroni corrections were performed to examine the differences in the access to improved water sources and improved sanitation facilities. Three sets of binary logistic regressions were also conducted on data from 2000, 2006, and 2011 to examine the associations between the access to improved water sources and improved sanitation facilities and household characteristics (geographical regions, wealth index, ethnicity of household head, sex of household head, and educational level of household head).

A dichotomous variable, ‘water sources and sanitation facilities in combination’, was derived whereby: 1 if a household had both ‘improved water sources AND improved sanitation facilities’, and 0, if a household had ‘unimproved water source OR unimproved sanitation facilities’ and ‘unimproved water sources AND unimproved sanitation facilities’. The results of these binary logistic regressions were reported as odds ratios (OR) with 95% confidence intervals (CI). STATA statistical software 12.0 (Stata Corporation) was used to perform all statistical analyses. The level of statistical significance was set at 0.05.

## Results

### Household trends in the access to water sources and sanitation facilities in Vietnam, 2000–2011

Data from the three survey rounds showed that the proportion of households with access to improved water sources in Vietnam has increased dramatically from 68.5% in 2000 to 90.4% in 2011 (Table 1). An increasing trend was also observed for sanitation facilities during this period (*p*<0.001). However, the proportion of households with access to improved sanitation facilities was lower than that for households with access to improved water sources, being 41.8% in 2000 and 77.5% in 2011 (Table 1).

**Table 1:** Access to improved water sources and improved sanitation facilities in Vietnam in 2000, 2006, and 2011

			Time		
			
			2000	2006	2011
Access to improved water sources^a^		*n*	5,224	7,351	10,735
		%	68.5	88.0	90.4
Access to improved sanitation facilities^a^		*n*	3,191	5,157	9,201
		%	41.8	61.7	77.5
Water sources and sanitation	Unimproved water sources AND	*n*	1,853	721	702
facilities in combination^a^	unimproved sanitation	%	24.3	8.6	5.9
	Improved water sources AND	*n*	2,640	4,873	8,764
	improved sanitation	%	34.6	58.3	73.8
	Unimproved water sources OR	*n*	3,135	2,762	2,408
	unimproved sanitation	%	41.1	33.1	20.3
Total		*n*	7,628	8,356	11,874
		%	100	100	100

a The differences in the proportions for 2000, 2006, and 2011 are statistically significant.

When we assessed water sources and sanitation facilities in combination, the households with access to unimproved water sources or unimproved sanitation facilities fell over time, from 41.1% in 2000 to 20.3% in 2011 (*p*<0.001, Table 1). We also observed an increasing trend in the proportion of households with access to both improved water sources and improved sanitation facilities, from 34.6% in 2000 to 73.8% in 2011 (*p*<0.001, Table 1).

### Household access to improved water sources and sanitation facilities in combination by region

**Fig. 1:**
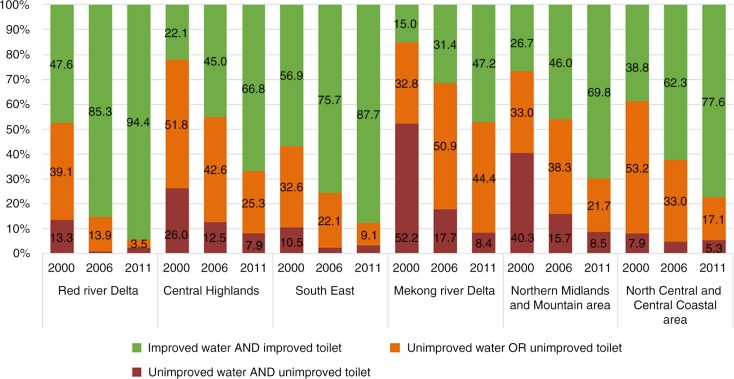
Proportion of households with access to improved water and sanitation facilities in 2000, 2006, and 2011, by regions. Chi-squared and Bonferroni method tests were used to compare the differences between regions and between time periods in each region.

Regarding access to *unimproved water sources or unimproved sanitation*, the proportions varied across the three survey rounds and the six regions (see orange graph in Fig. 1). In 2000, the highest level was observed in the North Central and Central Coastal Area (53.2%), and the lowest proportion was observed in the South East (32.6%) (*p*<0.05). From 2000 to 2006, the proportions decreased in four regions, that is, the Central Highlands (42.6%), North Central and Central Coastal Area (33%), South East (22.1%) and Red River Delta (13.9%) regions. The proportions, however, increased in the Mekong River Delta to 50.9% and in the Northern Midlands and Mountain area to 38.3%. In 2011, these proportions decreased dramatically compared to those reported in 2000, except for the Mekong River Delta, which increased from 32.8% in 2000 to 44.4% in 2011 and was the region with highest proportion of households still using unimproved water sources or unimproved sanitation facilities in 2011. The lowest proportion was in the Red River Delta (3.5%).

For households with access to *improved water sources and improved sanitation facilities in combination*, the proportions improved significantly in all regions, across the three surveys, although there were differences among regions (*p*<0.001) (see green graph in Fig. 1). In 2011, the highest proportion of households with improved water sources and improved sanitation facilities in combination was observed in the Red River Delta (94.4%) and the lowest proportion was in the Mekong River Delta (47.2%).

### Household access to improved water sources and improved sanitation facilities in combination by living areas

**Fig. 2:**
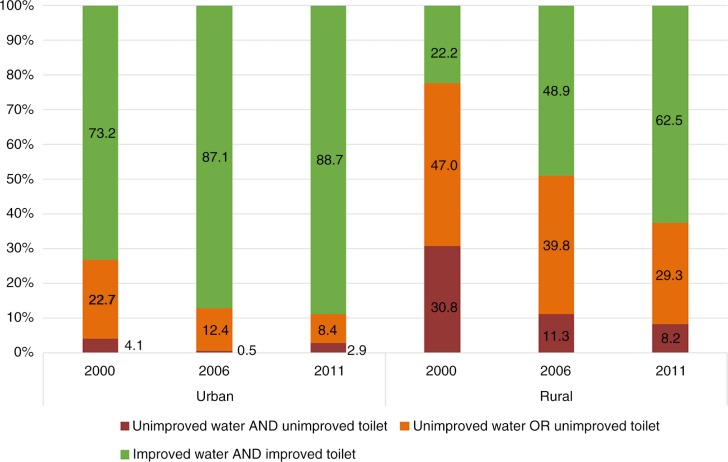
Proportion of households with access to improved water and sanitation facilities in 2000, 2006, and 2011, by living areas. Chi-squared and Bonferroni method tests were used to compare the differences between areas and between time periods in each area.

As described above, during the 12-year period from 2000 to 2011, we observed a dramatically increasing trend in households with access to improved water sources and improved sanitation facilities. However, as shown in Fig. 2, the proportions were statistically significantly higher in urban areas than in rural areas (*p*<0.01). For urban areas, the proportions of households with access to unimproved water and unimproved sanitation in the three surveys were low (under 5%) (see red graph in Fig. 2), while for rural areas, the proportion was high in 2000 (30.8%) but fell dramatically in 2011 (8.2%).

A similar trend was observed for the proportions of households with access to unimproved water sources or unimproved sanitation facilities (see orange graph in Fig. 2). The proportion in urban areas was 22.7% in 2000, but this fell significantly in 2011 to 8.4%. The proportion in rural areas was much higher, with 47% in 2000 and 29.3% in 2011 (*p*<0.001).

The proportion of households in urban areas with access to improved water sources and improved sanitation facilities increased from 73.2% in 2000 to 88.7% in 2011. However, the proportion in the rural areas was much lower, being 22.2% in 2000 and 62.5% in 2011. By 2011, the proportion in rural areas was still significantly lower than that in urban areas in 2000 (see green graph in Fig. 2, *p*<0.01). However, within rural areas, the increment was about 40% between 2000 and 2011.

### Household access to improved water sources and sanitation facilities in combination by wealth quintiles

**Fig. 3:**
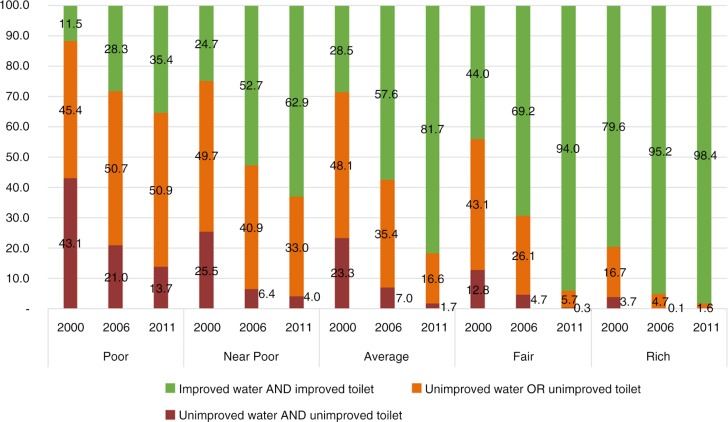
Proportion of households with access to improved water and sanitation facilities in 2000, 2006, and 2011, by wealth quintiles.

Figure 3 shows clear trends in increasing access to improved water sources and improved sanitation facilities across the three surveys rounds, as well as by wealth quintiles. In 2011, although 73.8% of households in Vietnam had access to both improved water sources and improved sanitation facilities, the proportion in the poorest quintile was just 35.4%. This means that about two thirds of households living in poor socio-economic conditions did not have access to improved water and improved sanitation facilities.

### Factors associated with the access to improved water sources and improved sanitation facilities

Table 2 gives the results of the multivariable logistic regressions for access to improved water sources and sanitation facilities in combination. Different factors were strongly associated with the access. There were some stark differences between regions. For example, in 2000, households from the Red River Delta region were more likely than those from the Mekong River Delta (the reference region), to have access to improved water sources and improved sanitation facilities (OR: 7.6; 95% CI: 5.8–10.0). In 2006, the OR increased to 17.4 (95% CI: 13.8–22.0) and 26.4 (95% CI: 20.0–34.9) in 2011. However, in the other regions, ORs (compared with the Mekong River Delta) either decreased or remained the same across the three survey years.

**Table 2:** Multivariable logistic regression of factors associated with household access to improved water sources and sanitation facilities in combination^a^, 2000, 2006, and 2011

		Odds ratio (95% confidence interval)		
		
		2000	2006	2011
Geographical regions	Central Highlands	2.5 (1.8–3.3)	3.5 (2.8–4.4)	3.7 (3.1–4.4)
	South East	4.8 (3.7–6.3)	4.0 (3.2–5.1)	4.8 (3.9–5.9)
	Northern Midlands and Mountain area	10.5 (7.9–14.0)	14.2 (11.0–18.4)	9.4 (7.7–11.5)
	North Central and Central Coastal area	7.7 (6.0–10.0)	7.1 (5.8–8.7)	5.9 (5.0–7.1)
	Red River Delta	7.6 (5.8–10.0)	17.42 (13.8–222)	26.4 (20.0–34.9)
	Mekong River Delta	1	1	1
Living areas	Urban	5.1 (4.4–6.0)	2.2 (1.8–2.6)	2.2 (1.9–2.5)
	Rural	1	1	1
Wealth index	Rich	11 (8.7–13.9)	25.0 (18.2–34.3)	42.3 (29.8–60.0)
	Fair	1.9 (1.6–2.3)	1.7 (1.5–2)	2.4 (2.1–2.8)
	Average	2.2 (1.8–2.7)	2.4 (2.0–2.8)	5.4 (4.5–6.4)
	Near poor	3 (2.5–3.7)	4.6 (3.8–5.5)	16.21 (13–20.2)
	Poor	1	1	1
Ethnicity of household head	Kinh	4.1 (3.3–5.2)	4.6 (3.7–5.6)	3.5 (2.9–4.1)
	Non-Kinh	1	1	1
Sex of household head	Female	1.1 (1.0–1.3)	1.1 (1.0–1.3)	1.2 (1.0–1.4)
	Male	1	1	1
Educational levels of household head	Secondary education or higher	1.5 (1.3–1.8)	4.3 (3.4–5.4)	1.8 (1.5–2.3)
	Primary education	1	2.4 (1.9–3.0)	1.3 (1.1–1.6)
	None	NA^b^	1	1

a The reference category is: using both unimproved water sources AND unimproved sanitation facilities or using unimproved water sources OR unimproved sanitation facilities

b in 2000, there was no surveyed household with no educational level; therefore, the group with ‘Primary education’ level was chosen as the reference group.

Households living in urban areas were more likely to have access to improved water sources and improved sanitation facilities in 2000 (OR: 5.1; 95% CI: 4.3–6.0). However, this likelihood fell in 2006 (OR: 2.2; 95% CI: 1.8–2.6) and in 2011 (OR: 2.2; 95% CI: 1.9–2.5). Therefore, the inequality in access to both improved water sources and sanitation facilities in combination between rural and urban areas decreased over the study period.

However, the wealth-based inequality in access to improved water sources and sanitation facilities increased during the study period. In 2000, rich households were over 11 times more likely to have access to improved water sources and improved sanitation facilities compared to the poor households (OR: 11.0; 95% CI: 8.7–13.9), and these odds increased over the study years. In 2011, households in the highest compared with the lowest wealth quintile were over 40 times more likely (OR: 42.3; 95% CI: 29.8–60.0) to have access to improved water sources and sanitation facilities in combination.

Table 2 also shows that in 2000, Kinh and/or Hoa (Chinese) ethnic people were more likely to have access to improved water sources and sanitation facilities than ethnic minority groups (OR: 4.1; 95% CI: 3.3–5.2). The OR increased slightly in 2006 (OR: 4.6; 95% CI: 3.7–5.6) and fell to 3.5 in 2011 (95% CI: 2.9–4.1). The educational level of the household head was also associated with the access to improved water sources and sanitation facilities in combination. Household heads with primary or higher education levels (secondary education or higher) were more likely to have access to improved water sources and sanitation facilities compared to household heads with no education (Table 2).

## Discussion

Based on our findings, great improvements have been made in providing access to improved water and sanitation facilities in Vietnam from 2000 to 2011. These data showed that by 2011, the proportions of the households with access to improved water and improved sanitation facilities had reached 90.4 and 77.5%, thus meeting the MDG's target for water and sanitation (88 and 75% by 2015) (4). However, the distribution of Vietnamese households with access to improved water sources and improved sanitation facilities in combination varied across the regions, living areas, wealth index quintiles, and ethnic groups.

In 2011, only 47% of households had access to both improved water sources and sanitation facilities in the Mekong River Delta region while in households in the Red River Delta region, 94% of households has access. Furthermore, households in the highest wealth quintile were over 40 times more likely (OR: 42.3; 95% CI: 29.8–60.0) to have access to safe drinking water and basic sanitation in combination, compared with the lowest wealth quintile. The level of inequality in access to both improved water sources and sanitation facilities increased between the wealth indices over time. Also, in 2011, households in the urban areas were twice as likely (OR: 2.1; 95% CI: 1.9–2.5) to have access to improved water sources and sanitation facilities in combination, compared to rural areas, and so were the Kinh ethnic groups.

In addition, it should be noted that when considering access to improved water sources and sanitation facilities in combination, 26.2% of the households in 2011 had no access to both ‘improved water sources and improved sanitation facilities’. This represents a high risk for feco-oral transmission and waterborne diseases, such as cholera, diarrhea, and dysentery in Vietnam. In fact, the limited access to clean water and hygienic sanitation accounted for 0.4% of the total disease-adjusted life years and 0.51% of the total deaths in the country in 2008 (10). Water scarcity in Vietnam could be due to physical water scarcity (a result of inadequate natural water resources to meet the national demand, including the dry conditions in the Central Highlands), or the economic water scarcity (a result of poor management of the sufficiently available water resources as in the Mekong River Delta region). Projections made by the United National Development Program warned of an expected increase in water shortages by 50% in the developing world by 2025, due to both population and industrial growth (11). This may make matters worse regarding efforts to increase access to improved water sources, especially for households in the Mekong River Delta and South East and Central Highlands regions, as well as for households with the lowest wealth.

The overall proportion of household access to improved sanitation facilities in Vietnam in the MICS 2011 was 77.5%, which was higher than that in Eastern Asia (67%) and the rest of the world (64%) (12). However, access to improved water sources and sanitation facilities was considerably lower in the Mekong River Delta region, Northern Midlands and mountainous areas, North Central and Central Coastal areas, rural areas, and for the poorest households. We also observed an increased level of disparity in the access to both improved water sources and sanitation facilities between the rural and urban areas, as well as among the six regions.

The impacts of water scarcity and the poor access to improved sanitation facilities are well recognized as causes of various infectious diseases (13). A recent study showed that diarrheal disease is the second leading cause of death in children under 5 years old (14). Almost half of the people living in the developing world have one or more diseases or infections (diarrhea, schistosomiasis, trachoma, and intestinal helminthes) attributed specifically to unsafe water, poor sanitation, and the lack of hygiene (15). In 2008, there were 2,785 mortality cases attributed to contaminated water and poor hygienic practices in Vietnam (10), and it was estimated that there were still approximately 2 million people in the country who still practiced open defecation in 2012 (16). Improving access to clean water and sanitation is essential in reducing the incidence of diseases in Vietnam. Esrey et al. (17) showed that in settings where the preexisting conditions were poor, a reasonably well-implemented intervention in one or more aspects of hygiene, sanitation, water supply, or water quality reduced the prevalence of diarrheal diseases up to one third. Also, water piped to one or more taps on a property could have greater reductions (up to 63%). Meanwhile, Sobsey et al. (18) identified the ceramic and bios household water filters to be the most effective methods. These water treatment methods in the household level also have the greatest potential to become widely used and be sustainable for improving the household water quality (18).

Although this study provided a comprehensive analysis of the current situation regarding access to improved water sources and sanitation in Vietnam, the data availability was confined to the three survey years 2000, 2006, and 2011. If the data sets for the surveys in 1995 and 2014 had been available, we could have explored a longer trend and made a more up-to-date assessment of the water and sanitation situation in the country.

##  Conclusions

Access to both improved water and sanitation in Vietnam from 2000 to 2011 was greatly increased and met the MDG's target 7C for water and sanitation. Categories for location and socio-economic characteristics such as regions, living areas, wealth indices and specific characteristics of the household's heads (ethnicity, gender, and educational levels) had a strong association with the access to improved water and sanitation facilities. The results of this analysis suggest that more efforts should be made to increase the access to improved water sources and sanitation facilities in the households of underserved populations, including those living in the Mekong River Delta, South East and Central Highlands regions. There is also a need to address the socio-economic factors that are associated with inadequate access to improved water and sanitation facilities. This will help ensure equal access to the basic needs and play an important role in the prevention and reduction of the burden of waterborne diseases and other diseases with fecal–oral transmission.
